# Interferon-Gamma Assay in Combination with Tuberculin Skin Test Are Insufficient for the Diagnosis of Culture-Negative Pulmonary Tuberculosis

**DOI:** 10.1371/journal.pone.0107208

**Published:** 2014-09-15

**Authors:** Marcin Wlodarczyk, Wieslawa Rudnicka, Beata Janiszewska-Drobinska, Grzegorz Kielnierowski, Magdalena Kowalewicz-Kulbat, Marek Fol, Magdalena Druszczynska

**Affiliations:** 1 Department of Immunology and Infectious Biology, Institute of Microbiology, Biotechnology and Immunology, Faculty of Biology and Environmental Protection, University of Lodz, Lodz, Poland; 2 Regional Specialised Hospital of Tuberculosis, Lung Diseases and Rehabilitation, Tuszyn, Poland; University of Cape Town, South Africa

## Abstract

**Objective:**

Early diagnosis of infectious cases and treatment of tuberculosis (TB) are important strategies for reducing the incidence of this disease. Unfortunately, traditional TB diagnostic methods are time-consuming and often unreliable. This study compared the accuracy and reliability of the tuberculin skin test (TST) and interferon (IFN)-γ-based assay (IGRA) for the diagnosis of active pulmonary TB Polish cases that could or could not be confirmed by *M. tuberculosis* (*M.tb*) culture.

**Methods:**

In total, 126 adult patients with clinically active TB or non-mycobacterial, community-acquired lung diseases (NMLD) hospitalised at the Regional Specialised Hospital of Tuberculosis, Lung Diseases and Rehabilitation in Tuszyn, Poland were enrolled in the present study. Sensitivity, specificity, positive predicted value (PPV), negative predicted value (NPV), and analytic accuracy (Acc) of TST and IGRA testing for the diagnosis of culture-positive and culture-negative TB patients were calculated. The quantities of IFN-γ produced in the response to *M.tb* specific antigens (TB Ag – Nil) in the cultures of blood from patients with active TB and NMLD patients were also analysed.

**Results:**

The IGRA sensitivity in culture-positive and culture-negative TB patients was similar, measuring 65.1% and 55.6%, respectively. The sensitivity of TST did not differ from the parameters designated for IGRA, measuring 55.8% in culture-positive and 64.9% in culture-negative TB. The sensitivity of TST and IGRA was age-dependent and decreased significantly with the age of the patients. No differences in the frequency or intensity of *M.tb*-stimulated IFN-γ production, as assessed by IGRA testing between culture-positive and culture-negative TB were noticed. Significantly lower concentrations of IFN-γ were observed in patients with advanced TB forms compared with those with mild or moderate TB pathologies.

**Conclusions:**

Our results do not show that a combination of IGRA and TST might be a step forward in the diagnosis of culture-negative TB cases. However, *M. tuberculosis*-stimulated IFN-γ levels might help to assess the extent of pulmonary TB lesions.

## Introduction

Tuberculosis (TB) remains a major worldwide cause of morbidity and mortality. It is estimated that one-third of the global population is infected with *Mycobacterium tuberculosis (M.tb)*, the causative agent of TB. The global burden of TB continues to be enormous. In 2012, there were an estimated 8.6 million new cases of TB, and 1.3 million people died from the disease [1]. Mortality is particularly high in those co-infected with human immunodeficiency virus (HIV) and multi-drug-resistant (MDR), extensively-drug-resistant (XDR) or totally-drug-resistant (TDR) *M.tb* strains.

The rapid detection of mycobacteria and successful treatment of contagious patients is important for controlling and preventing TB. Despite the considerable advances in TB diagnostic methodologies, the diagnosis of active disease still has serious limitations. The microscopic detection of acid-fast bacilli in clinical specimens or culture confirmation is frequently not possible. The diagnosis of patients with smear-negative and culture-negative results is complicated and is often based on clinical suspicion and appropriate response to anti-*M.tb* treatment.

One of the oldest diagnostic tests still in use worldwide is the tuberculin skin test (TST), also known as intradermal Mantoux test, which measures the delayed type hypersensitivity (DTH) response to a purified protein derivative (PPD) of *M.tb*. The test has variable sensitivity and specificity depending on the cut-off used and population screened [2]. A major limitation of the TST is the lack of specificity due to cross-reactivity with antigens present in other mycobacterial species, including the *M. bovis* Bacillus Calmette-Guerin (BCG) vaccine strain [3], [4], [5].

The identification of *M.tb*-specific immunodominant antigens encoded by the regions of difference (RD) 1 and 11 of the mycobacterial genome has led to the development of new diagnostic tests for *M.tb* infection. Interferon-gamma release assays (IGRA) are based on the release of interferon-γ (IFN-γ) in blood samples after *in vitro* stimulation with *M.tb*-specific proteins (ESAT-6, the early secretory antigenic target 6 kDa; CFP-10, the culture filtrate protein 10 kDa; TB7.7, Rv2654). These antigens are absent from all BCG strains and from most environmental mycobacteria [6], [7]. Two IGRA tests that incorporate RD1-specific antigens are available commercially: QuantiFERON-TB Gold In-Tube assay (QFT-IT) (Cellestis Limited, Carnegie, Australia) and T-SPOT.TB (Oxford Immunotec Limited, Abingdon, UK). Numerous studies have evaluated the utility of these tests in diagnosing active TB and have demonstrated a wide range of both specificity and sensitivity [8], [9], [10], [11].

In this study, we analysed the results of TST and IGRA testing (QuantiFERON-TB Gold In Tube) among 126 adult patients admitted with a clinical diagnosis of pneumonia to the Regional Specialised Hospital of Tuberculosis and Lung Diseases in Tuszyn, Poland. The analysis was performed in three groups of lung disease patients: 1, TB patients with positive *M.tb* sputum culture (culture-positive TB); 2, TB patients with negative *M.tb* sputum culture (culture-negative TB); 3, non-mycobacterial, community acquired lung diseases (NMLD) patients without any previous TB history.

## Materials and Methods

### Ethics statement

The study was conducted according to the principles expressed in the Declaration of Helsinki and was approved by the Ethics Committee of the Medical University in Lodz, Poland. Written informed consent from all patients was obtained before blood sampling.

### Study subjects

In total, 126 adult patients hospitalised at the Regional Specialised Hospital of Tuberculosis, Lung Diseases and Rehabilitation in Tuszyn, Poland, were enrolled in the present study between January 2010 and June 2011. The baseline information for all patients is shown in Table 1. All study subjects were examined and evaluated by infectious disease consultants. All of the patients underwent standard clinical and radiological examinations. Sputum samples were collected from the patients at the time of enrolment on three consecutive days, and were examined for acid fast bacilli (AFB) using Ziehl-Neelsen staining and subjected to mycobacterial culture using Lowenstein-Jensen medium. On admission to the hospital 3 ml of blood was taken for the IGRA assay, prior to the TST performance and the start of the treatment. The definitive diagnosis of lung disease was established after 8 weeks from the admission. This allowed dividing the patients into the following three groups: group 1, consisting of 43 patients diagnosed with pulmonary TB confirmed by positive *M.tb* sputum culture (culture-positive TB); group 2, consisting of 37 patients with pulmonary TB with negative *M.tb* sputum culture, but diagnosed on the basis of typical clinical symptoms, typical features' on radiographs and proper responses to anti-TB treatment (culture-negative TB); group 3, consisting of 46 patients with excluded TB, suffering from non-mycobacterial, community acquired lung diseases (NMLD), treated and cured with wide-range antibiotics (i.e. amoxicillin/clavulanic acid, clarithromycin, clindamycin, ceftriaxone, ciprofloxacin, doxycyline), with triple negative *M.tb* sputum culture and no previous TB history. According to the medical data, all studied subjects were BCG vaccinated and had negative results on serological tests for human immunodeficiency virus (HIV) infection. One person from the NMLD group suffered from sarcoidosis. Six patients, three from each TB group had a past history of cured pulmonary TB. Demographic, clinical, radiological and microbiological data were collected for all patients at the time of enrolment. The data included BCG vaccinations, any history of previous TB infection or anti-tuberculous treatment, and any other underlying diseases (i.e., malignant diseases, diabetes mellitus, cardiovascular disease, neurologic disease, and chronic renal failure).

**Table 1:** Demographic characteristics of TB and NMLD patients studied.

	Group of patients		
	Pulmonary TB		NMLDb)
	culture-positive	culture-negativea)	n = 46 (%)
	n = 43 (%)	n = 37 (%)	
Age (yrs)	48.6±18.2	51.7±15.5	52.7±17.3
Sex			
Female	21 (48.8)	21 (56.8)	30 (65.2)
Male	22 (51.2)	16 (43.2)	16 (34.8)
Ethnicity	Caucasians	Caucasians	Caucasians
BCG vaccination	43 (100)	37 (100)	46 (100)
Smoking history	26 (61.9)	19 (57.6)	10 (34.5)
Alcohol abuse history	11 (26.2)	7 (21.2)	3 (10.0)
Past history of TB	3 (7.0)	3 (8.1)	0 (0)
Radiological findings			
Extent of lesionc)			
1 (TB mild)	16 (37.2)	12 (32.5)	
2 (TB moderate)	10 (23.3)	10 (27.0)	not applicable
3 (TB advanced)	17 (39.9)	15 (40.5)	
Cavity (+)	20 (46.5)	17 (45.9)	
Infiltration shadow (+)	21 (48.8)	21 (56.8)	
Underlying disease			
Malignant disease	4 (9.3)	3 (7.5)	5 (10.9)
Diabetes mellitus	5 (11.6)	2 (5.4)	2 (4.3)
Cardiovascular disease	4 (9.3)	2 (5.4)	8 (17.4)
Neurologic disease	1 (2.3)	0 (0)	0 (0)
Chronic renal failure	1 (2.3)	1 (2.7)	0 (0)

a) TB sputum culture negative in triple testing.

b) NMLD, non-mycobacterial community acquired lung diseases.

c) Data denote the extents 1, within one-third of the unilateral lung field (TB mild) 2, within the unilateral lung field (TB moderate) and 3, beyond the unilateral lung field (TB advanced).

### Tuberculin skin test (TST)

The tuberculin skin test was performed using 2 tuberculin units (TU) of purified protein derivative (PPD) RT23 (Statens Serum Institute, Copenhagen, Denmark) and the Mantoux technique at the beginning of the treatment for all patients diagnosed. The diameter of skin induration was measured after 48–72 h by experienced staff. A result was considered positive when the size of the induration was ≥10 mm.

### Interferon-gamma release assay (IGRA)

The IGRA assay was performed using the QuantiFERON®-TB Gold In-Tube (QFT-IT) kit (Cellestis Ltd., Carnegie, Australia) according to the manufacturer's instructions. In brief, a total of 3 ml of blood was taken from each patient and collected in 3 tubes of 1 ml each (Nil control, TB antigen-specific (TB Ag), Mitogen control). Following a 24 hour incubation at 37°C, the tubes were centrifuged (2500 RCF, 15 min), and the concentration of IFN-γ in the harvested plasma was measured by ELISA. The optical density (OD) of each sample was measured using a multifunctional counter Victor 2 (Wallac Oy, Turku, Finland) fitted with a 450 nm filter. The data were processed and interpreted using the calculation QuantiFERON-TB Gold Analysis Software supplied with the kit. The test result was considered to be positive if the IFN-γ level in the sample tube after stimulation with TB Ag was ≥0.35 IU/ml (after subtraction of the value for the Nil tube). The quantitative IFN-γ concentration data obtained by IGRA testing were also calculated and analysed in the study.

### Statistical analysis

Statistical analysis was performed using the Statistica 10.0 PL software program (Statsoft). Categorical data were compared using the χ-squared test or Fisher's exact test. Comparisons between the IFN-γ concentrations were made by the Kruskal-Wallis Anova test. A p value of less than 0.05 was considered significant. Concordance between the results of the TST and QFT-IT tests was assessed using κ coefficients, where κ values above 0.8 indicate excellent agreement, values of 0.8–0.4 represent good correlation, and values below 0.4 indicate poor agreement. Sensitivity, specificity, positive predicted value (PPV), negative predicted value (NPV), and analytic accuracy (Acc) were calculated for each TB patient group. The analysis was performed in patients with culture-positive or culture-negative active tuberculosis and subjects with non-mycobacterial lung diseases, characterized by clinical symptoms, triple negative sputum culture and cured with standard wide-range antibiotics. Confidence intervals (95% CI) were estimated according to the binomial distribution. A cut-off level of IFN-γ measured by QFT-IT ELISA was determined by receiver operator characteristic (ROC) curve analysis using the MedCalc software (Belgium).

## Results

### Study subjects characteristics

The study population consisted of 80 (42 females, 38 males) Polish patients diagnosed with active pulmonary TB, microbiologically confirmed (43) or not confirmed (37) by a triple sputum culture and of 46 (30 females, 16 males) patients with nonmycobacterial lung diseases (NMLD) without TB history who had negative results on the triple sputum culture. All of the patients were recruited at the Regional Specialised Hospital of Tuberculosis and Lung Diseases in Tuszyn, Poland. The main demographic characteristics of TB and NMLD patients included in the study are shown in Table 1. The mean age of the patients in each group was similar, at 48.6±18.2; 51.7±15.5; 52.7±17.3 years, respectively. The radiological examination of the lungs in culture-negative and culture-positive TB patients showed no differences in the extent of the lesions or the presence of cavities or infiltration shadows (Table 1). Based on the extent of the lesions in their lung tissue, TB patients were classified as having (1) mild (lesions within one-third of the unilateral lung field), (2) moderate (lesions within the unilateral lung field) or (3) advanced (lesions beyond the unilateral lung field) TB disease. Similar proportions of culture-positive and culture-negative TB patients were included in groups 1, 2 and 3. Three out of 43 (7%) patients with culture-positive TB and 3 out of 37 (8.1%) culture-negative TB patients had a past history of healed pulmonary TB. The frequency of underlying diseases did not differ between the studied TB patient groups. Four out of 43 (9.3%) culture-positive and 3 out of 37 culture-negative TB patients suffered from malignant diseases. Diabetes mellitus was diagnosed in 5 (11.6%) and 2 (5.4%) culture-positive and culture-negative TB patients, respectively. Similar percentages of bacteriology-positive and bacteriology-negative patients with TB suffered from cardiovascular diseases (9.3% vs. 5.4%) and chronic renal failure (2.3% vs. 2.7%). One person from the NMLD group was diagnosed with sarcoidosis.

### TST and IGRA results

The tuberculin skin test was performed for all patients and the diameter of skin induration was measured 48–72 hours after intradermal administration of PPD. Taking into account the moderate exposure to *M.tb* in Poland as well as the BCG vaccination schedule based on repeated anti-tuberculous immunizations of TST-negative children and adolescents (compulsory from 1955 to 2006), in our study a skin induration of at least 10 mm was considered a positive reaction to tuberculin. TST was positive in 56% (24/43) and 65% (24/37) of culture-positive and culture-negative TB patients, respectively (Table 2). In contrast, positive reactions to PPD with a diameter of skin induration greater than 10 mm were developed only by 28% (13/46) NMLD patients (p<0.05). A strong positive response to PPD with a skin induration diameter of more than 15 mm was observed in 11 (26%) TB culture-positive, 14 (38%) TB culture-negative, and 8 (17%) NMLD patients. The proportion of TST positive results when using the cut off ≥5 mm was 56% (24/43) or 65% (24/37) in culture-positive or culture-negative TB patients and 37% (17/46) in NMLD subjects and it did not differ substantially from the proportion defined as positive using a 10 mm cut off. TST positivity was defined as an induration ≥10 mm with the respect to the highest age group. In our study the sensitivity of TST significantly decreased with the age of the diagnosed patients.

**Table 2:** TST and IGRA results in TB and NMLD patient groups.

Patients	TST* result		IGRA result	
	n (%)		n (%)	
	-	+	-	+
Pulmonary TB				
culture-positive	19 (44%)	24 (56%)	15 (35%)	28 (65%)
culture-negative	13 (35%)	24 (65%)	16 (44%)	20 (56%)
NMLD	33 (72%)	13 (28%)a)	40 (87%)	6 (13%)b)

*TST induration cut-off 10 mm.

a) Significantly different NMLD patients from culture-positive (p = 0.008) and culture-negative (p = 0.009) TB patients.

b) Significantly different NMLD patients from culture-positive (p = 0.00001) and culture-negative TB patients (p = 0.00001).

In culture-positive and culture-negative TB groups a similar prevalence of positive IGRA results was found (65% vs 56%). One out of 37 (2.7%) culture-negative TB patients had an indeterminate IGRA result. The frequency of IGRA positives was significantly lower among NMLD (13%) than TB patients (p<0.05). All 3 culture-positive TB patients with cured TB were characterized with positive TST and positive IGRA results. However, among culture-negative TB patients 1 person was TST-positive/IGRA-positive, two other patients TST-positive/IGRA-negative and TST-negative/IGRA-negative.

Analysis of the results of skin reactions to tuberculin with a 10 mm cut-off in three age ranges of TB patients (<30 years, 30–59 years, >60 years) showed a negative correlation between the age and development of DTH to the subcutaneously injected PPD (p = 0.02; r = −0.34). The percentage of tuberculin-positive culture-positive TB patients decreased from 72% in the youngest group to 61% and 39% in the groups of 30–59 and >60 years, respectively (Table 3). A similar tendency was noticed in the group of culture-negative TB patients, among whom the percentage of positive TST decreased from 100% in the patients under 30 to 50% in people over 60 years old. Such a trend was not observed in NMLD patients, where the frequency of positive TST was similar across the entire group (25%, 32% and 21% in each age category, respectively, data not shown). The analysis of IGRA results in the age groups showed a decrease in the rate of IGRA positives for culture-positive TB patients, from 100% in the youngest group to 61% and 54% in the groups of 30–59 and >60 years, respectively, but not for TB culture-negative patients (Table 4).

**Table 3:** Sensitivity, specificity, positive predictive value, negative predictive value and analytic accuracy of TST in culture-positive or culture-negative TB patients.

TB patients	Age	TST* result						
		n (%)		Sensitivity	Specificity	PPV	NPV	Acc
		-	+	*(95% CI)*	*(95% CI)*	*(95% CI)*	*(95% CI)*	*(95% CI)*
culture-positive	total	19	24					
		(44%)	(56%)	55.8%	71.7%	64.9%	63.5%	64.0%
								
	<30	2	5	71.4%	75.0%	83.3%	60.0%	72.7%
		(28%)	(72%)	*(38.0*–*104.9)*	*(32.6*–*117.4)*	*(53.5*–*113.2)*	*(17.1*–*102.9)*	*(41.9*–*103.6)*
	30–59	9	14	60.9%	67.9%	60.9%	67.9%	64.7%
		(39%)	(61%)	*(40.9*–*80.8)*	*(50.6*–*85.2)*	*(40.9*–*80.8)*	*(50.6*–*85.2)*	*(48.4*–*81.0)*
	>60	8	5	38.5%	78.6%	62.5%	57.9%	59.3%
		(61%)	(39%)	*(12.0*–*64.9)*	*(57.1*–*100.1)*	*(29.0*–*96.0)*	*(35.7*–*80.1)*	*(35.2*–*83.3)*
culture-negative	total	13	24					
		(35%)	(65%)	64.9%	71.7%	64.9%	71.7%	68.7%
								
	<30	0	4	100.0%	75.0%	80.0%	100.0%	87.5%
		(0%)	(100%)	*(100.0*–*100.0)*	*(32.6*–*117.4)*	*(44.9*–*115.1)*	*(100.0*–*100.0)*	*(63.0*–*112.0)*
	30–59	8	15	65.2%	67.9%	62.5%	70.4%	66.7%
		(35%)	(65%)	*(45.8*–*84.7)*	*(50.6*–*85.2)*	*(43.1*–*81.9)*	*(53.1*–*87.6)*	*(50.8*–*82.5)*
	>60	5	5	50.0%	78.6%	62.5%	68.8%	66.7%
		(50%)	(50%)	*(19.0*–*81.0)*	*(57.1*–*100.1)*	*(29.0*–*96.0)*	*(46.0*–*91.5)*	*(43.6*–*89.8)*

*TST induration cut-off 10 mm.

TST, tuberculin skin test; PPV, *positive predicted value*; NPV, *negative predicted value*; Acc, *analytic accuracy*; CI, *confidence interval*.

**Table 4:** Sensitivity, specificity, positive predictive value, negative predictive value and analytic accuracy of IGRA in culture-positive or culture-negative TB patients.

TB patients	Age	IGRA result						
		n (%)		Sensitivity	Specificity	PPV	NPV	Acc
		-	+	*(95% CI)*	*(95% CI)*	*(95% CI)*	*(95% CI)*	*(95% CI)*
culture-positive	total	15	28					
		(35%)	(35%)	65.1%	87.0%	64.9%	72.7%	76.4%
								
	<30	0	7	100.0%	100.0%	100.0%	100.0%	100.0%
		(%)	(100%)	*(100.0*–*100.0)*	*(100.0*–*100.0)*	*(100.0*–*100.0)*	*(100.0*–*100.0)*	*(100.0*–*100.0)*
	30–59	9	14	60.9%	89.3%	82.4%	73.5%	76.5%
		(39%)	(61%)	*(40.9*–*80.8)*	*(77.8*–*100.7)*	*(64.2*–*100.5)*	*(58.7*–*88.4)*	*(63.2*–*89.8)*
	>60	6	7	53.8%	78.6%	70.0%	64.7%	66.7%
		(46%)	(54%)	*(26.7*–*80.9)*	*(57.1*–*100.1)*	*(41.6*–*98.4)*	*(42.0*–*87.4)*	*(44.9*–*88 4.)*
								
culture-positive	total	16	20					
		(44%)	(56%)	65.1%	87.0%	64.9%	72.7%	76.4%
								
	<30	2	3	60.0%	100.0%	100.0%	66.7%	77.8%
		(40%)	(60%)	*(17.1*–*102.9)*	*(100.0*–*100.0)*	*(100.0*–*100.0)*	*(28.9*–*104.4)*	*(47.0*–*108.6)*
	30–59	9	11	55.0%	89.3%	78.6%	73.5%	75.0%
		(45%)	(55%)	*(33.2*–*76.8)*	*(77.8*–*100.7)*	*(57.1*–*100.1)*	*(58.7*–*88.4)*	*(60.9*–*89.1)*
	>60	5	6	54.5%	78.6%	66.7%	68.8%	68.0%
		(46%)	(54%)	*(25.1*–*84.0)*	*(57.1*–*100.1)*	*(35.9*–*97.5)*	*(46.0*–*91.5)*	*(45.8*–*90.2)*

IGRA, interferon-gamma release assay; PPV, *positive predicted value*; NPV, *negative predicted value*; Acc, *analytic accuracy*; CI, *confidence interval*.

**Figure 1:**
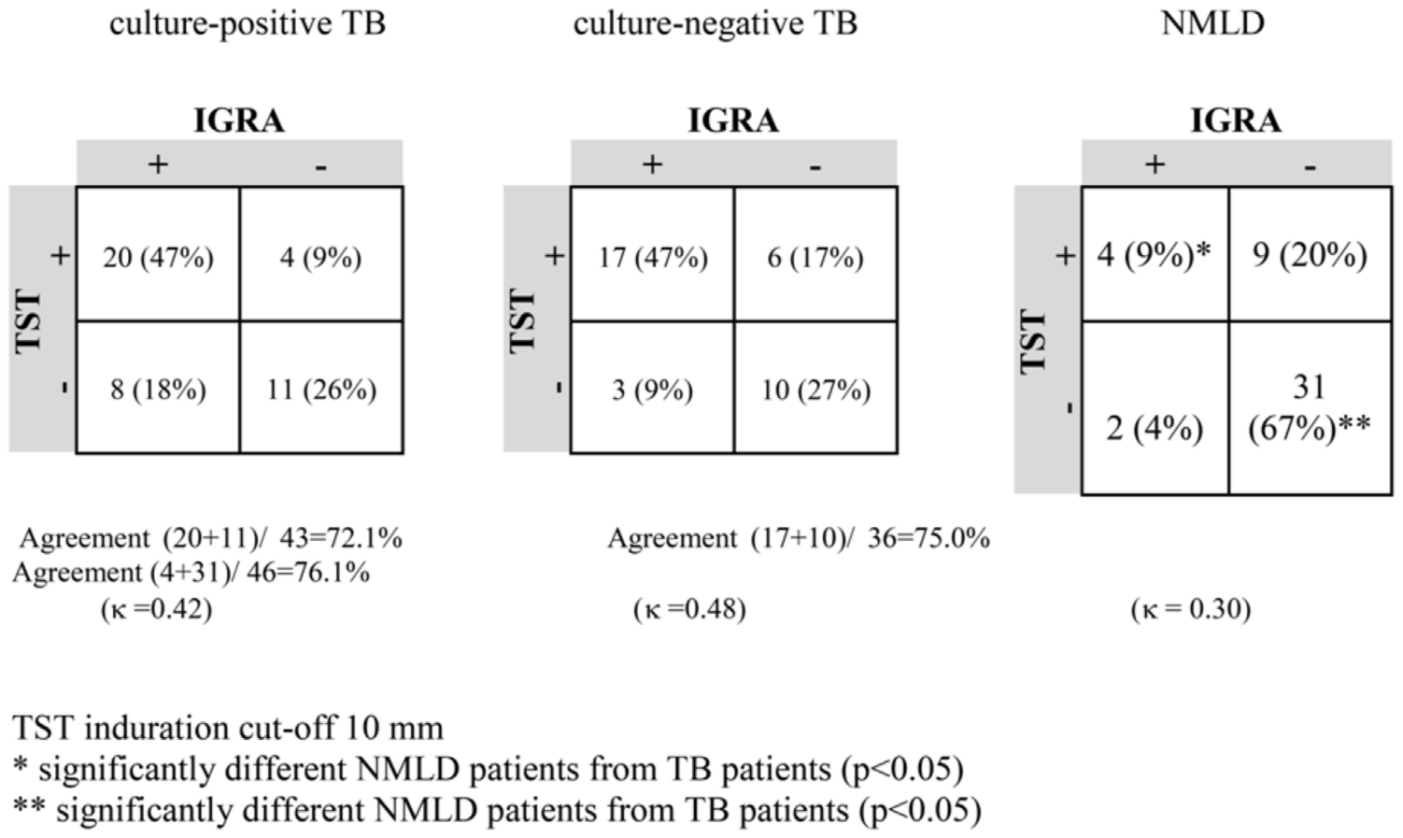
Agreement between the TST and IGRA results.

The overall agreement between the TST and IGRA in all patients was good (κ = 0.48). The TST and IGRA concordance rate in both TB groups was also good (κ = 0.42 and κ = 0.48), although this value was poor in NMLD patients (κ = 0.30) (Fig. 1). The rate of positive results for both TST and IGRA was significantly higher among TB patients (47%) than NMLD subjects (9%) (p<0.05) (Fig. 1). In contrast, 67% NMLD patients vs. 26% TB patients had negative TST and IGRA results (p<0.05). No difference was found in the rate of discordant results of TST and IGRA between the studied groups of TB patients (p>0.05). The overall sensitivity, specificity, analytic accuracy, positive and negative predictive values for the TST and IGRA were similar in both of the studied TB groups (Table 3, Table 4).

The diagnostic parameters of skin TST in culture-positive TB were 55.8%, 71.7%, 64.0%, 64.9% and 63.5%, respectively, whereas those for culture-negative TB patients were 64.9%, 71.7%, 68.7%, 64.9% and 71.7%, respectively (Table 3). The corresponding values for IGRA testing were 65.1%, 87%, 76.4%, 64.9% and 72.7% in culture-positive and 55.6%, 87%, 73.2%, 76.9% and 71.4% in culture-negative TB patients, respectively (Table 4).

### IFN-γ responses to *M.tb* specific antigens

The quantities of IFN-γ produced in the response to *M.tb* specific antigens (TB Ag – Nil) in the cultures of blood from patients with active TB confirmed or not confirmed by sputum culture and NMLD patients were also analysed. The cut-off value for the ELISA assay was determined by the receiver operating characteristic (ROC) curve analysis (MedCalc) performed in two groups (1) culture-positive TB patients and (2) subjects with non-mycobacterial lung diseases, characterized by clinical symptoms, triple negative sputum culture and cured with standard wide-range antibiotics.

**Figure 2:**
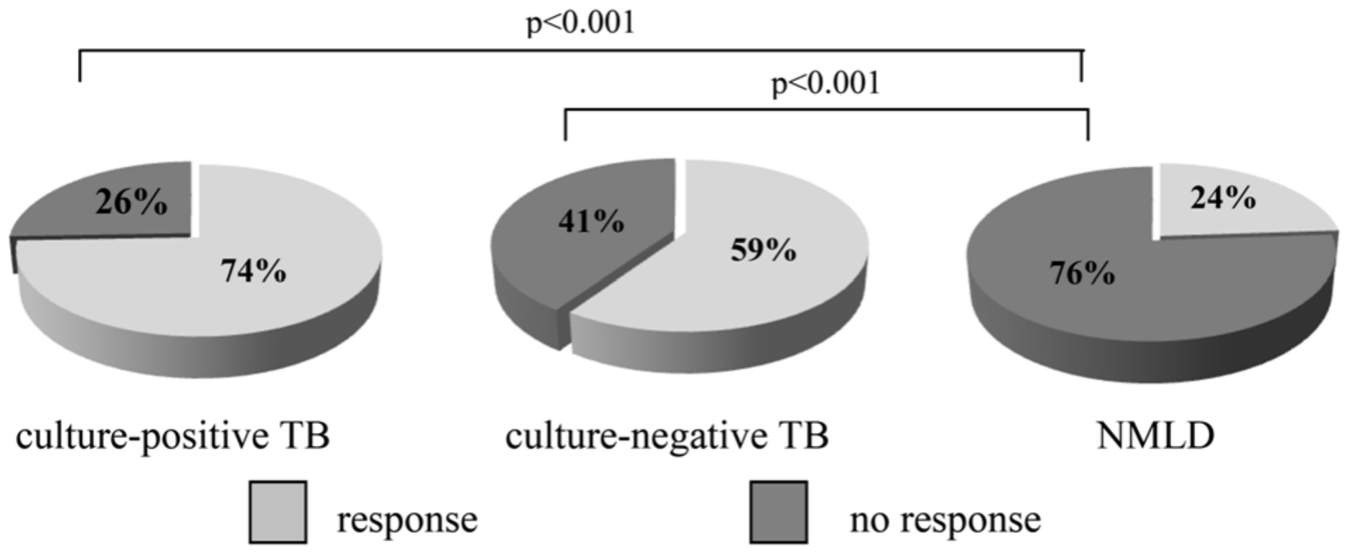
The frequency of IFN-γ response to *M.tb* specific antigens.

As shown in Fig. 2, the frequency of the IFN-γ response to *M.tb* antigens in culture-negative and culture-positive TB patients was similar, at 74% and 59%, respectively (Fig. 2). However, there was a significant difference in the frequency of IFN-γ production in whole blood cultures between TB and NMLD patients. Leukocytes from both the culture-positive and culture-negative TB patient groups responded to mycobacterial antigens by IFN-γ secretion significantly more frequently than those of the NMLD volunteers (24%) (p<0.001).

**Figure 3:**
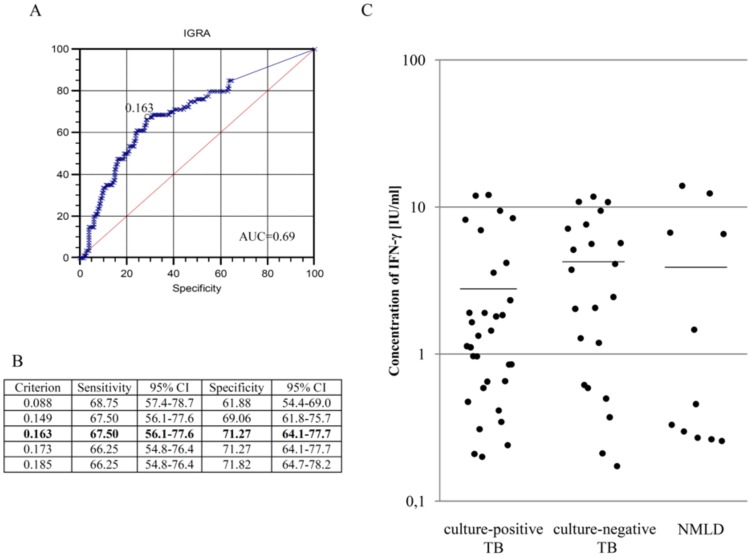
The mean quantity of IFN-γ produced in response to the *M.tb*-specific antigens used in IGRA. A.B. The cut-off value for a positive response was set at 0.163 IU/ml by receiver operator characteristic (ROC) curve analysis. C. Each dot represents individual response of one participant in the study and horizontal lines represent the mean values. The geometric mean ± SD for culture-positive TB was 2.78±3.50 IU/ml (n = 32), for culture-negative TB 4.24±3.86 IU/ml (n = 22) and for NMLD 3.89±5.19 IU/ml (n = 11).

The threshold value set by the ROC analysis was 0.163 IU/ml (Fig. 3A). The sensitivity and specificity of the ELISA assay corresponding to the cut-off of 0.163 IU/ml were 67.5% and 71.27%, respectively (Fig. 3B). No significant differences were noticed in the mean IFN-γ levels of those blood cultures stimulated with mycobacterial antigens from culture-positive (2.78±3.50 IU/ml) or culture-negative (4.24±3.86 IU/ml) TB patients or from patients with non-mycobacterial community acquired lung diseases (3.89±5.19 IU/ml) (Fig. 3C). Comparing the concentrations of the cytokines in both TB groups, the patients with advanced forms of TB were characterised by a lower IFN-γ level (culture-positive: 1.00±1.06 IU/ml, culture-negative: 1.38±1.44 IU/ml) than those with mild (culture-positive: 4.59±4.77 IU/ml, culture-negative: 5.92±4.19 IU/ml) or moderate (culture-positive: 3.45±3.49 IU/ml, culture-negative: 5.06±3.86 IU/ml) TB forms (Table 5).

**Table 5:** The production of IFN-γ in response to *M.tb* antigens in patients with mild, moderate or advanced TB form.

TB form	TB					
	culture-positive			culture-negative		
	IFN-γ (IU/ml)					
	responders/group	mean ± SD	p*	responders/group	mean ± SD	p*
	(%)			(%)		
mild	9/16 (56%)	4.59±4.77	0.02	10/12 (83%)	5.92±4.19	0.04
moderate	10/10 (100%)	3.45±3.49	0.12	6/11 (54%)	5.06±3.86	0.21
advanced	13/17 (76%)	1.00±1.06		7/15 (47%)	1.38±1.44	

* significance value between the intensity of IFN-γ responses in patients with advanced TB and mild or moderate TB form.

There were no significant differences in the frequency or intensity of IFN-γ production between culture-positive and culture-negative TB patients, with or without lung cavities and infiltration shadows (Table 6). The lowest concentrations of IFN-γ were noted in the blood cultures from patients with advanced TB, regardless of the prevalence of cavities and infiltrations into the lungs.

**Table 6:** The production of IFN-γ in response to *M.tb* antigens in culture-positive and culture-negative TB patients with or without lung cavities and infiltration shadows.

TB form	IFN-γ (IU/ml)											
	TB culture-positive						TB culture-negative					
	responders/group (%)	mean ± SD	p*	responders/group (%)	mean ± SD	p*	responders/group (%)	mean ± SD	p*	responders/group (%)	mean ± SD	p*
	cavities			no cavities			cavities			no cavities		
mild	4/4			5/12			4/5			5/7		
	(100%)	5.92±5.03	0.04	(42%)	3.53±4.83	0.15	(80%)	5.82±3.81	0.05	(71%)	5.98±4.92	0.39
moderate	7/7			3/3			2/5			4/5		
	(100%)	3.45±3.05	0.25	(100%)	3.44 ±5.19	1.0	(40%)	5.60±7.12	0.12	(80%)	4.41±4.76	1.0
advanced	8/9			5/8			2/7			5/8		
	(89%)	1.32±1.26		(62%)	0.49±0.31		(28%)	2.30±2.54		(62%)	1.02±0.95	
	infiltration shadows			no infiltration shadows			infiltration shadows			no infiltration shadow		
mild	6/7			3/9			6/7			3/5(		
	(86%)	4.42±4.59	0.29	(33%)	4.94±6.18	0.10	(86%)	5.56±4.33	0.28	60%)	6.63±4.72	0.13
moderate	6/6			4/4			2/5			4/5		
	(100%)	3.66±3.30	0.66	(100%)	3.12±4.26	0.51	(40%)	6.12±1.41	0.35	(80%)	4.52±4.79	0.62
advanced	6/8			7/9			5/9			2/6		
	(75%)	0.65±0.47		(78%)	1.30±1.36		(56%)	1.77±1.56		(33%)	0.41±0.28	

* significance value between the intensity of IFN-γ responses in patients with advanced TB and mild or moderate TB.

**Figure 4:**
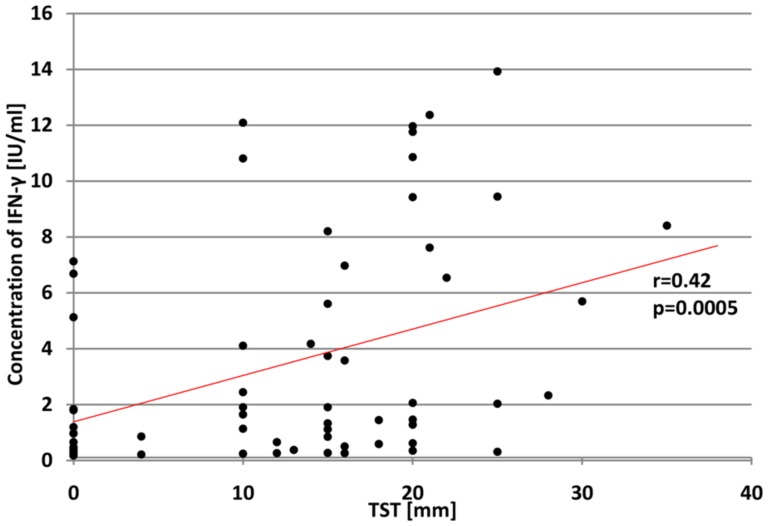
The correlation between IFN-γ levels produced in response to the *M.tb*-specific antigens and TST size in TB patients' group.

The statistical analysis of the relationship between IFN-γ concentrations in *M.tb*-stimulated blood cultures from TB patients and of the DTH to the subcutaneously injected PPD showed that the level of the cytokines increased with the diameter of skin induration (r = 0.42, p = 0.0005) (Fig. 4).

## Discussion

Despite all efforts, TB remains a major global problem. Vaccination with Bacille Calmette-Guerin (BCG) confers good protection against disseminated childhood TB but provides variable protection against pulmonary disease, especially in adolescents and adults. The duration of the BCG protection remains debatable, although there is good evidence that the protection declines with time [12]. Thus, better TB control can only be achieved by a combination of an effective vaccination strategy and faster and better diagnosis for treating individuals with TB. Unfortunately, the traditional methods of TB diagnosis are not only time consuming but also unreliable. Sputum smear examination has a sensitivity of only 50%, whereas sputum culture can confirm pulmonary TB in around 80% of cases [13]. Although patients with sputum smear-negative or culture-negative TB may be less infectious than patients with bacteriology-confirmed TB, they also contribute to TB transmission. Direct light microscopy can detect mycobacteria in the sputum at a minimum density of 5000–10000 AFB per ml of specimen, whereas the infectious dose is only a few mycobacterial cells. Therefore, people in contact with culture-negative TB patients are also at risk of *M.tb* infection and subsequent development of active TB disease. Methods based on direct nucleic acid testing have been used to facilitate the diagnosis of pulmonary TB in people with suspected TB who have repeated negative sputum smears and cultures. The sensitivity and specificity estimates for nucleic-acid amplification tests for the diagnosis of TB in respiratory specimens are highly variable, with sensitivities lower and more inconsistent than specificities [14]. A number of barriers exist with respect to the widespread use of genetic diagnostic assays, including the costly specialised equipment and trained personnel. Inadequate tools and the failure of conventional microscopy and cultures in the laboratory diagnosis of active TB have contributed to the underdiagnosis of the disease, leading to individual morbidity and mortality and to continued transmission [15]. Most cases of bacteriology-unconfirmed TB are diagnosed on the basis of clinical features, radiological findings, tuberculin skin testing and interferon-gamma release assays, whose practical value has been undermined.

Our results demonstrated that the overall sensitivity of IGRA observed in culture-negative and culture-positive TB patients was similar, at 55.6% and 65.1%, respectively. Similar sensitivities were demonstrated in the studies of Winqvist et al. (52%) Dewan et al. (60%) and Lui et al. (60%) [16], [17], [18]. The specificity of the interferon test (87%) was also similar or even higher than that reported in numerous publications [9], [19], [20], [21]. Discrepancies in the IGRA sensitivity and specificity found in our study and by others could be explained by differences in the ethnic origins, ages or immune statuses of the patients. As we showed in our work, the IGRA sensitivity was age-dependent and a higher sensitivity of the test was observed among younger patients than those aged older than 60 years. In contrast, Kobashi et al. [22] observed no difference in the rate of positive IGRA results between younger and older TB patients in Japan. However, the sensitivity of IGRA is known to differ in populations with either various prevalences of TB or a policy of BCG immunisation at an early age [23]. Furthermore, the sensitivity of IFN-γ responses to *M.tb*-specific antigens was shown to decrease significantly in HIV-infected and immunocompromised patients [24], [25]. The value of the TST in the diagnosis of active pulmonary TB has been contested. A major limitation of the test is the cross-reactivity of the PPD with antigens present in BCG bacilli and most environmental mycobacteria. As a test measuring an immunologic response, it demonstrates a decreasing sensitivity with increased immune deficiency. There are many false-negative results among patients with immune dysfunctions or false-positive results in patients who exhibit sensitivity to tuberculin after BCG vaccination or infection with mycobacteria other than the *M.tb* complex. Many countries continue to use BCG vaccination as part of their TB control program, particularly in infants. The WHO recommends that BCG should be given to all children born in countries highly endemic for TB. Poland introduced mandatory BCG immunization in 1955. Currently, in Poland BCG vaccine is administered to newborn babies once - on the first day of life. However, up to 2006 Polish children and adolescents were vaccinated with BCG more than once, on the first day of life, at the age of 6, 12 and often 18 if TST performed at that time was negative. The criteria for interpreting the response to BCG vaccination estimated on the basis of the skin reaction to PPD vary depending upon the strain of BCG used, the risk of *M.tb* infection and characteristics of the person being tested. Based on the sensitivity and specificity of TST, the prevalence of TB and mandatory BCG immunization in Poland, a diameter of skin induration of at least 10 mm was considered to be a positive reaction to tuberculin. In our study the overall sensitivity and specificity of TST with a cut-off of 10 mm for patients with culture-positive and culture-negative TB did not differ from the parameters designated for IGRA. Similarly to IGRA, the sensitivity of the TST significantly decreased with the age of the diagnosed patients. Similarly, in a Japanese study, the rates of positive TST results were significantly higher in younger (70%) compared to elderly (27%) patients [22]. These data show that age-associated changes in the activity of the immune system must be taken into account in the evaluation of diagnostic, prophylactic or therapeutic proceedings. Accumulating data confirm an inverse relationship between immune status or general health, and response to vaccination or longevity, suggesting that the immune system becomes less effective with advancing age [26]. Impaired function of the immune response in aged individuals may explain the increased susceptibility of elderly individuals to infectious diseases, including TB.

It is well-accepted that cell-mediated immunity (CMI), especially the type-1 T-cell response that is associated with the production of IFN-γ, plays a key role in controlling *M.tb* infections [27]. During the initial phase of the infection, antigens secreted by the replicating mycobacteria are presented to T cells by infected antigen-presenting cells, and generating a strong CMI response. IFN-γ is mainly produced by antigen-specific effector T cells that have encountered antigens and can rapidly respond with the cytokine production. Because the frequency of effector T cell response was shown to be associated with the antigen load, the amount of produced IFN-γ might be associated with the bacterial load in the infected host. Therefore it seems that the measurement of *M.tb*-specific type-1 T-cell responses may represent a sensitive method of detecting early TB infections, even predicting progression to active disease [28].

Our study did not show any differences in the *M.tb*-stimulated IFN-γ levels between culture-negative and culture-positive TB patients. However, we found the IFN-γ response to be decreased in patients with advanced TB forms, compared to those with mild or moderate TB pathologies. Our results are consistent with the previous reports showing a decrease in IFN-γ responses related to an increased severity of pulmonary TB [29], [30]. Moreover, Hasan et al demonstrated decreased ESAT-6-induced IFN-γ production in patients with severe disseminated TB in comparison to those with spinal, meningeal or abdominal TB forms [31]. Increased IFN-γ responses in less severe TB infections correlated with a greater frequency of antigen-specific CD4 T cells in *M.tb*-infected individuals with a low bacterial burden, compared with patients with a higher bacterial load. In animal models, the T-cell responses to both ESAT-6 and crude *M.tb* culture filtrate correlated with the progression of TB and the level of bacterial replication *in vivo* [32], [33], [34]. A direct correlation between the ESAT-6-stimulated IFN-γ levels two weeks after infection and the bacterial numbers in the lungs 6 weeks after infection was demonstrated. At an early stage of *M. bovis* infection in cattle, Buddle et al. [35] suggested that enhanced IFN-γ responses to ESAT-6 could predict the development of progressive TB disease. The results were confirmed by Vordermeier et al. [36], who demonstrated a correlation between the ESAT-6-stimulated level of IFN-γ and the degree of pathology in *M. bovis*-infected cattle. Andersen et al. [28] proposed that a TB-specific increase in the IFN-γ response might be useful for identifying individuals with progressive *M.tb* infections who are likely to develop active TB disease. The authors suggested that, in addition to a standard assessment of the IGRA test results, it is possible to establish a cut-off value that will identify those individuals in advanced stages of the infection who are most at risk of developing active contagious TB. Doherty et al. [37] observed a high level of IFN-γ response to ESAT-6 correlating with the subsequent development of active TB in healthy household contacts of TB patients and suggested that rising concentrations of this cytokine might serve as a marker of TB prognosis.

In conclusion, the present study demonstrated that despite many diagnostic possibilities the diagnosis of culture-negative TB cases is still difficult and ambiguous. The IGRA assay was not more sensitive than TST, and its surprisingly low positive predictive value (PPV) confirmed the necessity of using it in combination with other methods for the diagnosis of active TB. False negative results are too frequent to propose this immunological testing to clinicians in order to diagnose active tuberculous disease. Symptoms, radiological pictures and the effect of antituberculous therapy still remain more useful in the case of culture-negative TB. The commercialized IGRA and TST seem to be more helpful in the diagnosis of latent forms of TB. Nethertheless, *in vitro* diagnostic tests for *M.tb* infection based on the detection of soluble IFN-γ in whole blood cultures incubated with specific mycobacterial antigens might also help to differentiate between mild and advanced TB and to assess the extent of pulmonary TB lesions, however the individual context of the patients should always be considered.
